# The global burden of teacher burnout: Evaluating the roles of workload and social support in diverse educational contexts

**DOI:** 10.1192/j.eurpsy.2025.1469

**Published:** 2025-08-26

**Authors:** A. El Alaiki, F. Hadrya, Z. Boumaaize, H. Guider, M. A. Lafraxo, A. Soulaymani, A. Mokhtari, H. Hami

**Affiliations:** 1 Laboratory of Biology and Health, Faculty of Science, Ibn Tofail University, Kenitra; 2 University Hassan First of Settat, Higher Institute of Health Sciences, Health Sciences and Technologies Laboratory, Settat; 3 Higher Institute of Nursing Professions and Health Techniques, Oujda, Morocco

## Abstract

**Introduction:**

Teacher burnout is a pervasive challenge across the global educational sector, profoundly impacting educator well-being and the overall quality of education. A robust body of research highlights the link between organizational factors and burnout, underscoring the urgent need for an in-depth understanding of these dynamics across different cultural contexts.

**Objectives:**

This systematic review aims to delineate how workload and social support dynamically influence teacher burnout. Through a detailed examination of the complex interrelationships among these factors, we endeavor to elucidate the underlying mechanisms by which they influence teachers’ emotional and psychological health, thereby providing critical insights into potential evidence-based interventions.

**Methods:**

We synthesized findings from a total of 40 relevant studies (35 cross-sectional and five longitudinal studies), adhering to the 2020 PRISMA guidelines. We utilized the Newcastle-Ottawa Scale for quality assessment due to its rigorous criteria, examining the impact of workload and social support on teachers’ stress levels across diverse educational settings.

**Results:**

High workload and insufficient social support were identified in 75% of the studies as significant predictors of emotional exhaustion among teachers globally. Excessive workloads were correlated with increased burnout levels, which negatively affected their mental health and job satisfaction. Conversely, strong social support networks, including collegial relationships and administrative support, were found to effectively mitigate burnout, bolstering teachers’ resilience and overall well-being. Furthermore, the review underlined that the quality of the evidence was moderate, highlighting the need for further, more robust research.

**Conclusions:**

This review confirms the complex interactions within organizational dynamics that contribute to teacher burnout. It underscores the critical need for tailored interventions, such as professional development in stress management and policies that foster supportive work environments. By strategically addressing workload challenges and enhancing social support, stakeholders can significantly improve teacher well-being and reduce burnout risks globally.

**Disclosure of Interest:**

None Declared

